# Systems biology platform for efficient development and translation of multitargeted therapeutics

**DOI:** 10.3389/fsysb.2023.1229532

**Published:** 2023-09-18

**Authors:** Karim Azer, Irina Leaf

**Affiliations:** Axcella Therapeutics, Cambridge, MA, United States

**Keywords:** drug discovery, drug development, disease modelling, metabolomics, platform, systems biology, systems pharmacology

## Abstract

Failure to achieve efficacy is among the top, if not the most common reason for clinical trial failures. While there may be many underlying contributors to these failures, selecting the right mechanistic hypothesis, the right dose, or the right patient population are the main culprits. Systems biology is an inter-disciplinary field at the intersection of biology and mathematics that has the growing potential to increase probability of success in clinical trials, delivering a data-driven matching of the right mechanism to the right patient, at the right dose. Moreover, as part of successful selection of targets for a therapeutic area, systems biology is a prime approach to development of combination therapies to combating complex diseases, where single targets have failed to achieve sufficient efficacy in the clinic. Systems biology approaches have become increasingly powerful with the progress in molecular and computational methods and represent a novel innovative tool to tackle the complex mechanisms of human disease biology, linking it to clinical phenotypes and optimizing multiple steps of drug discovery and development. With increasing ability of probing biology at a cellular and organ level with omics technologies, systems biology is here to stay and is positioned to be one of the key pillars of drug discovery and development, predicting and advancing the best therapies that can be combined together for an optimal pharmacological effect in the clinic. Here we describe a systems biology platform with a stepwise approach that starts with characterization of the key pathways contributing to the Mechanism of Disease (MOD) and is followed by identification, design, optimization, and translation into the clinic of the best therapies that are able to reverse disease-related pathological mechanisms through one or multiple Mechanisms of Action (MOA).

## 1 Background

Impressive advancements have been made in our ability to probe and investigate the genetic and molecular causes of diseases within the last few decades. High-throughput measurements such as epigenomics, proteomics, metabolomics, transcriptomics, and genomics have expanded our knowledge of living organisms and their internal structural components, including cells, tissues, and organ systems. The vision for fully characterizing the integrated cellular networks and *in silico* whole cell systems predictions is unfolding and has made enormous strides over the last two decades (Wierling et al., 2015a; Benson, 2015; Imam et al., 2015; Chen and Li, 2016; Malod-Dognin et al., 2019; Helwer and Chen, 2022; Zareifi et al., 2022). There is much work ahead, while today’s advancements have paved the way for important applications in drug and vaccine discovery and development.

**FIGURE 1:**
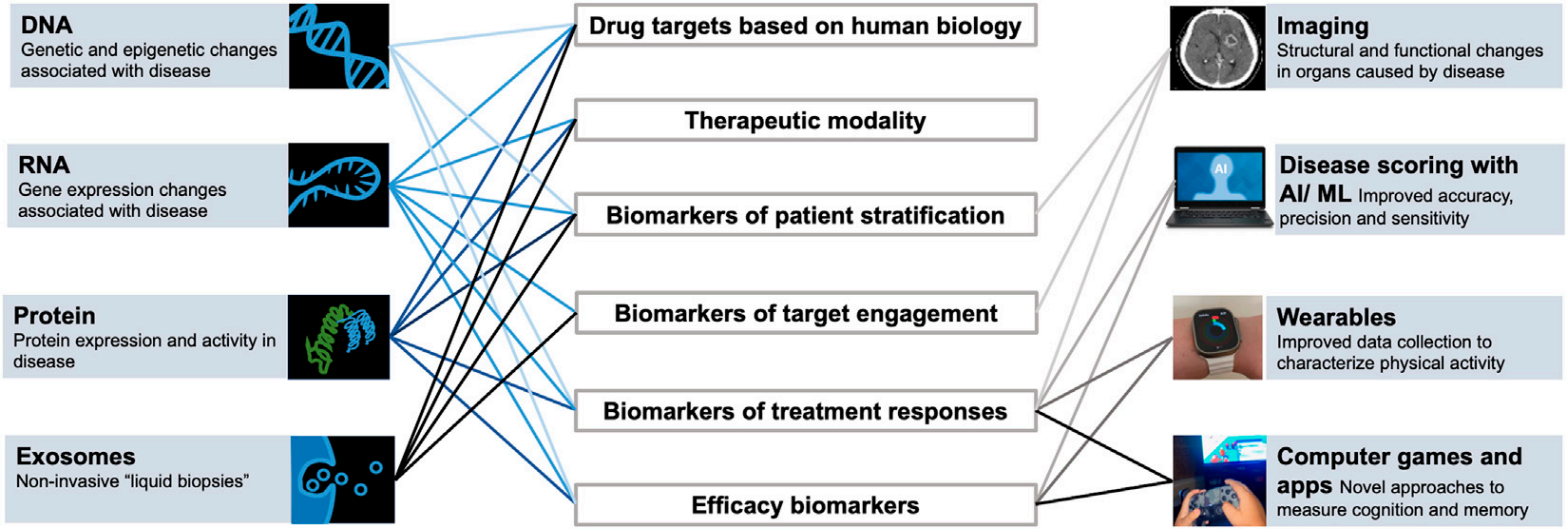
Technological advancements enable generation of new types of data and multimodal datasets to study biological systems and support drug discovery and development.

While “single target” drugs could work efficiently to affect a critical molecular component contributing to a specific disease mechanism, especially at early stages of disease onset, the same treatment approach for complex diseases remains unsatisfactory (Yan, 2010). The complexity of human biology makes it challenging to develop safe and effective drugs (Berg et al., 2005), often embodied by the complexity of the mechanism of disease (MOD) and in parallel mechanism of action (MOA) of a drug that makes the current drug discovery and development approach challenging (Kovatchev et al., 2009; Iyengar, 2013). Drug approvals for treating complex and multifactorial diseases have dwindled despite increased insights into disease mechanism and the availability of a large volume of data generated over past decades that is ready to be assembled, analyzed, and interpreted by various technological methodologies (Figure 1). System-wide regulation of the biological systems for both complex and rare genetic diseases is at play as evidenced by incomplete penetrance and disease heterogeneity even in genetic diseases with defined causal genetic mutations including cancers, Amyotrophic Lateral Sclerosis (ALS), Huntington’s, Parkinson, Phenylketonuria (PKU), Alpha-1 Antitrypsin Deficiency (AATD) where inheritance of causal disease mutations is not sufficient for developing a disease (Cooper et al., 2013; Turner et al., 2013; Shawky, 2014; Taeubner et al., 2018). It puts into question the concept of a single gene, single target hypothesis. With an increased understanding of pleiotropic mechanisms simultaneously contributing to pathological changes and disease progression across a wide spectrum of diseases, we are now seeing an evolution in drug development to consider combination therapy, as evidenced in areas like cancer, asthma, and others (Saleh, 2008; Iyengar, 2013; Obenauf, 2022; Plana et al., 2022). A novel patient- and resource-centric paradigm within the drug discovery and development approach are to minimize and reduce the costly “trial-and-error” methodology for novel drugs and drug combinations. Moreover, increased focus is afforded to molecular mechanism-based, targeted strategies that could address underlying mechanisms of disease, and engineer combinations with corresponding multi-targeted mechanisms of action that restore homeostatic processes and improve efficacy endpoints, when given at the right dose to the right patient. Advanced computational methods applied to multi-scale data including clinical and molecular patient profiles have a great potential to identify signatures for patient stratification in heterogeneous diseases and to identify patient subsets that are more likely to respond to the treatment. With commensurate advances in computational approaches, computer microchips and cloud computing scalability, the opportunity to learn and identify new and complex biological systems implicated in disease that can be re-programmed with novel therapeutic modalities is at hand.

Here, we describe how the field of systems biology, representative of a multidisciplinary approach to drug discovery and development, integrates biological, computational, and pharmacological sciences capabilities to innovatively identify, design, and translate novel molecular entities into the clinic against complex and difficult-to-treat diseases.

## 2 Systems biology evolution: role in drug discovery and development

The inherent complexity of human biological systems and the pathological perturbations leading to complex diseases holistically require a systematic approach that combines genetic, molecular, cellular, physiological, clinical, and technological methodologies to characterize disease heterogeneity and individualize drug discovery, development, and treatment paradigms (Silverman and Loscalzo, 2013). Biological systems are inherently a complex network of multi-scale interactions, as exemplified by emergent properties, and therefore inadequately represented or characterized by individual molecular components (Zou et al., 2013). A “single-target-based” drug development approach is notably less effective for complex diseases (Wierling et al., 2015b), with lower probability of success, and higher risk to address underlying disease biology, presenting a fundamental challenge in the current practice for drug discovery and development (Kovatchev et al., 2009). Close investigation of the multi-scale multi-target interactions of a disease network (MOD) and accurate mapping of the drug’s mechanism of action (MOA) are both critical for building confidence in the therapeutic hypothesis while potentially de-risking off-target effects and bracketing the therapeutic window (Tatonetti et al., 2009; Silverman and Loscalzo, 2013).

Addressing these complexities involves the integration of diverse, large-scale data types accessible from well-designed clinical registries and trials, preclinical studies, biomarker databases, gene and protein curated databases, and large, virtual compound libraries (Walters et al., 1998; Hert et al., 2006; Li et al., 2012; Wassermann et al., 2013), databases with documented biological, chemical, and structural activities of chemical compounds (Parsons et al., 2006; Wishart et al., 2006; Kuhn et al., 2008), biological information available via the human genome project (Lander et al., 2001) and understanding of cellular and molecular factors driving disease states using high-throughput screening techniques and network-based technologies (Giaever et al., 1999; Hughes et al., 2000; Lum et al., 2004; Parsons et al., 2004; Joyce and Palsson, 2006; Perlstein et al., 2007; Hillenmeyer et al., 2008; Hoon et al., 2008). Notably, over the past several decades, technological advances in biology research have generated a vast quantity of omics-related molecular datasets derived at the level of genomics (DNA sequencing, structure, function, mapping, and evolution of genomes), transcriptomics (RNA sequencing that allows to quantify gene expression changes at the organ or single cell level), proteomics (mass spectrometry and affinity based methods with significantly increased protein coverage that allow to quantify thousands of proteins in cells, tissues, or biofluids, and mapping of post-translational modifications), and metabolomics (unbiased and targeted panels for quantification of metabolites representing substrates and products of metabolism in cells, tissues or biofluids). Such an enormous amount of information at a multiscale level of organization affords a unique opportunity to laying the foundation for effectively decoding complex biological systems implicated in disease and deciphering the mechanism of disease (MOD). While these big data streams offer the unprecedented potential to discover and distill key components of the MOD, still some challenges remain as far as data fidelity and breadth, the incremental costs associated with experiments, ability to mine these complex data robustly and in a reproducible manner, and the translatability of preclinical models to the living human organs and systems in health and in disease.

The use and application of advanced mathematical models to study biological systems is increasing in drug development, informed by the increasing availability of informative data. The advent of innovative and large-scale computing technologies, computational methodologies, novel learning and prediction approaches like artificial intelligence, and cloud-based capabilities is rapidly closing the gap and ability to analyze and integrate voluminous datasets using various statistical and dynamical models that could advance our MOD or MOA understanding. Advanced computational methods applied to large preclinical and clinical datasets allow one to characterize and design successful clinical biomarker strategy for quantitative translation into the clinic that can enable patient stratification and selection for enrollment of the right patient subsets from the heterogeneous patient population, and detection of drug activity and early modulation of disease mechanisms predictive of beneficial changes in important efficacy endpoints and clinical outcomes for an early Go/No-Go decision making (Van’t Veer et al., 2002; Wang et al., 2005; Shen et al., 2009; Shen et al., 2012; Ali et al., 2014; Kantor et al., 2004; Mirnezami et al., 2012).

Systems biology is an inter-disciplinary field that applies computational and mathematical methods to the studies of complex interactions within biological systems as opposed to the traditional reductionist approach used in research (Hood and Tian, 2012). As a multidisciplinary field, at the intersection of biology, computation and technology, systems biology is geared towards leveraging omics technologies to investigate and quantify biology as a system or network (Berg et al., 2005; Wierling et al., 2015b; Zhou et al., 2019). Utilizing multi-modality datasets, the systems biology approach seeks to re-integrate critical elements to describe how multicomponent interactions form functional networks within an organism and/or patient and how their dysfunction contributes to a particular disease state (Bielekova et al., 2014). To date, systems biology methods contributed to generation of extensive bioinformatics tools including biological pathway maps and networks in health and disease built using data from preclinical models and human samples that can be integrated with quickly growing human genetics findings and other data types to strategically enable new drug discovery and identification of novel therapeutic adjacencies or indications for existing drugs (Qu and Rajpal, 2012; Wierling et al., 2015a; Obenauf, 2022; Zareifi et al., 2022).

With its increased role in drug discovery and development, the systems biology-based approach has evolved well into the translational and clinical space. This approach offers novel insights into complex diseases and corresponding drug discovery and development to enable design of novel combinations that could re-program disease biology with a prescribed MOA, the translation of preclinical findings into potential clinical benefits (Pujol et al., 2010), biomarker signature-based patient selection, advancement of disease biomarkers, prediction of drug response, elucidation of disease MOD and drug’s MOA, and drug re-purposing.

## 3 Convergence of computational methodologies and biological processes for informing disease models

In the past decade, there has been an increased convergence of computational and biological research, with closer collaborations that lead to better characterized disease models (Navlakha and Bar-Joseph, 2011). Biologists rely more than ever on computational sciences to study biology by analyzing and interpreting large data sets. At the same time, computational scientists and engineers rely on data generated to improve model performance, reliability, predictions, and validation. Moreover, researchers are motivated by high-level design principles of biological systems to inspire various computational methods. The increasing availability of systems-level data derived from advanced measurement technologies enables scientists to characterize the multi-scale nature of human biology and complex disease pathophysiology to inform MOD (Azer et al., 2021). Increased knowledge of the molecular pathway interactions and networks constituting the biological system function at the cell, tissue, organ, and organism level allows for investigation of the MOA of candidate molecules (Butcher et al., 2004; Hood and Perlmutter, 2004; Sobie et al., 2011). The combination of computational and biological sciences enables a data-driven scientific cycle of learning, refining, and confirming hypotheses. Thus, computational biology, is an invaluable tool in proposing a biological hypothesis for experimental validation (Komurov et al., 2012; Zhou et al., 2019).

With emerging systems and network approaches, experimental models can be utilized to provide a data-driven system-wide reconstruction of the interactive and dynamic changes in cellular and molecular components based on samples derived from cell-based assays, preclinical animal models or human studies. Multiple data types can be integrated using omics tools to construct cell signaling models, networks, and pathways to identify new drug targets and help discover novel leads by elucidating pathway mechanisms and components. Efficient utilization of the multi-omics datasets and data-driven discovery for preclinical models correlated with experimentally simulated disease phenotypes could be more impactful in identifying better compounds and drug discovery opportunities (Berg et al., 2005).

Nevertheless, utilizing model systems depends on their appropriateness and translatability to human disorders. The systems biology methodology integrates the human disease biology knowledgebase and combinatorial design that will allow design of better and more precise experimental models and phenotypes for investigation of molecular networks and MOD and for testing and selecting the best therapeutic compounds and combinations. E.g., the BioMAP^®^ system, a primary human cell-based assay designed to model complex human disorders in a functional *in vitro* format (Butcher, 2005; Unternaehrer and Daley, 2011). Stimulating primary human cell types and co-cultures using pathway activator combinations assists in generating cell signaling networks appropriate for human disorders. The systems biology approach is often utilized to select primary human cell types, pathway activator combinations, and endpoint and biomarker selection for these assays (Berg et al., 2005; Stacey, 2012).

Creating models against clinically established phenotypes and utilizing high-throughput drug screening capabilities could allow integration of the experimental results with disease databases to predict and prioritize new indications with the highest probability to benefit from selected treatments. These databases, e.g., human whole-cell models (Szigeti et al., 2018); and the Human Cell Atlas Project (Regev et al., 2017), will integrate an understanding of human biology and disease pathophysiology at the cellular scale and enable crucial information for mathematical biology, model development and application (Azer et al., 2021). Moreover, leveraging a significant array of existing models, e.g., through EMBL-EBI Biomodels Database (https://www.ebi.ac.uk/biomodels/) and tools such as SBML Toolbox (http://sbml.org/Downloads) enables a rapid and efficient model development process and allows for the advantages of open science platforms as the developed models are shared and built upon by the scientific community (Malik-Sheriff et al., 2020).

Advanced statistical learning approaches applied to large clinical and preclinical data could help better characterize the mechanisms of pertinent disease, MOD, and mechanisms of action of candidate drugs, MOA. Ultimately, using omics technology could help reduce the risk of “trial-and-error” in probing and predicting behavior of complex biological systems (Berg et al., 2005).

Application of systems biology is especially critical for complex diseases, considering their unknown etiology and the limited understanding of molecular mechanisms driving disease pathogenesis. Since complex, multifactorial disorders have substantial heterogeneity, selecting right patients for appropriate therapies based on the MOD/MOA hypothesis for such complex diseases cannot be achieved without the analysis of the large multi-modal datasets (genomic, epigenomic, proteomic and metabolomic) using next-generation computational methods (Silverman and Loscalzo, 2013). Systems biology analysis has been applied to defining segments of patients that are more likely to respond to targeted treatments in asthma based on their genomic profile or genetic variant subtypes (Karaaslan et al., 2022). This analysis has also been applied to advancement and discovery of biomarkers of disease and response such as alpha-synuclein for Parkinson (Siderowf et al., 2023) and neurofilament light chain (NfL) for several neurodegenerative diseases, and for advancing molecular characterization of MOD to facilitate the discovery of novel therapies, e.g., in Long COVID (Su et al., 2022).

Drug discovery and development decisions rely extensively on characterizing a target’s MOA, which is crucial in characterizing a drug’s pharmacologic effect (Berg et al., 2005) and its impact on biological processes and pathways. Data derived from experimental models focused on the MOA of a specific drug can be integrated with analysis of the real-world data from clinical disease registries and from available translational studies and clinical trial data to hone in on specific mechanisms implicated in complex diseases and identify clinical and molecular signatures associated with disease subtypes, for example, in non-alcoholic steatohepatitis (NASH), type 2 diabetes, and other complex diseases (Zhou et al., 2019; McGlinchey et al., 2022; Zareifi et al., 2022). Moreover, better understanding of a drug’s MOA builds more supportive evidence and confidence in the therapeutic hypothesis and allows for de-risking of clinical development, e.g., through identification of biomarkers of response, and advancing data-driven rationale for identifying drug-responders in heterogeneous disease populations.

Overall, systems biology approaches could help define phenotypes, timepoints, readouts, and biomarkers to target in the preclinical models during research stages of drug discovery and development by leveraging clinical registries and clinical trial data from relevant disease populations, and utilizing established clinical endpoints and biomarkers. Clinicians and investigators can draw on findings from both biological (*in vitro, in vivo and ex vivo* experiments) and mathematical models (*in silico* experiments) to decipher causal disease mechanisms and guide future decisions for better interventions.

## 4 Systems biology as a drug discovery and development engine

In this new era of technological and data sciences progress, systems biology approach has a great potential to improve and accelerate drug discovery and development process through efficient utilization of existing publicly available and new well-designed datasets representing preclinical and clinical transcriptomic, proteomic, metabolomic, imaging, and other data types. Advanced computational methods can be applied for the integration and analysis of the data from multiple sources and multiple formats, and for the generation of the data-driven therapeutic hypothesis that will be tested experimentally and *in silico* with subsequent parameter optimization for successful drug candidates and combinations.

**FIGURE 2:**
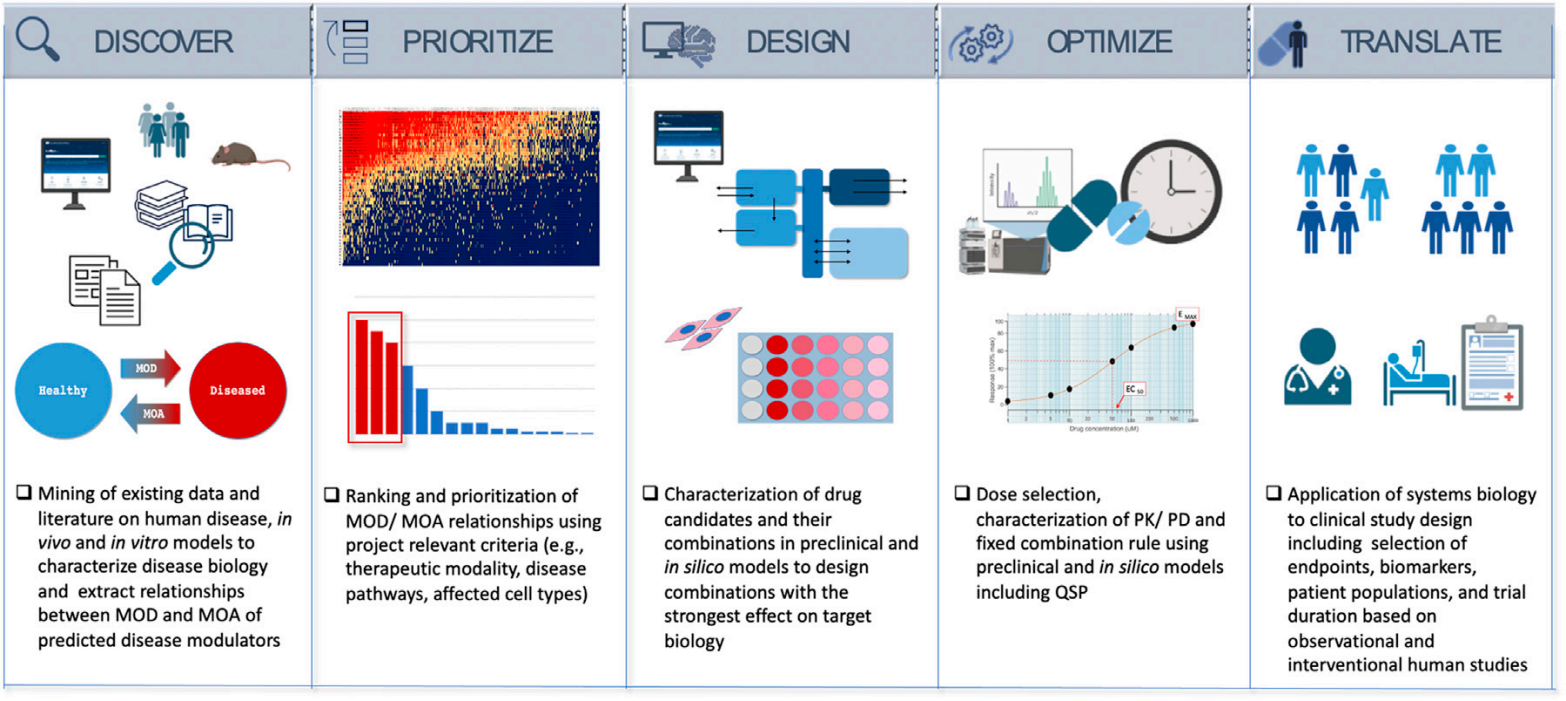
Systems biology as a drug discovery and development engine.

The following five steps provide a roadmap from the data-science driven discovery of disease mechanisms and potential therapies to the experimental validation, optimization of drug combinations and dose, and to informing successful clinical trial design (Figure 2).

• Discover—Characterize the Mechanism of Disease (MOD) and identify potential modulators to reverse disease biology and restore health (MOA).
• Prioritize/Rank—Rank the MOD and drug candidates/MOA predictions.
• Design—Select and confirm drug candidates that have the highest potential to affect the intended MOA, utilizing a combination of experimental and computational models.
• Optimize—Find the optimal composition, component ratios, and the dose to yield maximum treatment effect relevant for the clinical studies.
• Translate—Develop a clinical path to inform clinical study design and biomarker strategy to validate pharmacology and efficacy in the clinic.

### 4.1 Discover—investigate the mechanism of disease (MOD) and identify potential modulators to reverse disease biology and restore health

Mechanisms associated with disease biology (MOD) and drug candidates predicted to modulate disease via MOD-reversing mechanism (MOA), can be identified in this step by the integrative analysis of mechanistic data from multiple sources including scientific literature, internal and external datasets, relevant databases, pathway maps and networks using advanced data science approaches. Mining and assembly of the relevant data sources provides context and content for precedented or evaluated molecular mechanisms and targets for a given disease and the tools such as animal models or *in vitro* systems used to test novel candidates against these targets. Literature mining approaches including natural language processing (NLP), keyword-based methodologies, semantic-based and ontology-based searches, can be leveraged to gain insight into molecular mechanisms of diseases of interest (MOD) and match them with potential drug targets at a larger scale and with increased precision (Wu et al., 2012; Joseph et al., 2016; Azer et al., 2021). To investigate the biology of interest, specific research objectives and questions have to be established to customize the data sciences algorithms by designing screening principles, keywords, and criteria to be applied to selected data formats. Machine learning (ML) principles can be applied for automatic annotations and specific knowledge extraction tasks.

ML approaches can also be leveraged to integrate the data from multiple sources and to build disease networks for internal and external datasets such as experimental results, historical clinical data, and disease biomarker data (Parolo et al., 2021). The networks can be constructed *de novo* or can be overlayed onto pathways, e.g., KEGG (Kanehisa and Goto, 2000), or REACTOME (Gillespie et al., 2022). Perturbations of these networks by disease (MOD) and by potential therapeutic modulators (MOA) can be predicted, mapped, and analyzed in this step simultaneously for many pathways and drugs or compounds using cloud empowered *in silico* simulations (Clish et al., 2004; Valeyev et al., 2010; Ellen, 2014). These computational models are customized based on the project objectives, scope, modality of choice and other parameters. Computational workflows (Denaro et al., 2023) can be developed and streamlined to enable semi-automated and efficient analysis of big data, and to allow a broader team of scientists to analyze and interpret the data. Advanced computational tools like sensitivity analysis, asymptotic analysis, surface-response modeling and others can be leveraged to identify patterns and hone in on key variables driving network behaviors (Merrill et al., 2019; Simoni et al., 2021).

Overall, leveraging the literature, internal and external datasets coupled with data science approaches provides an opportunity for a reliable, data-driven knowledge discovery process of network model development for the biological systems of interest towards advancing an MOD strategy for a given disease and initial identification of potential molecular candidates predicted to reverse components of the MOD.

### 4.2 Prioritize—rank the MOD and drug candidates/MOA predictions

The advantage of the data sciences approach is the ability to digest large amounts of data and generate selected outputs at a large scale. In order to rank these outputs and prioritize and select the mechanisms and targets for subsequent follow-up and validation, the strategy needs to be established based on general and project-specific principles. The target product profile provides broad context, criteria, and direction for development. Some of the general principles for analyzing, interpreting, and ranking the processes and outputs are.

• the strength of evidence based on human genetics, clinical precedence, and preclinical experimental models.
• phenotypic characteristics and common biological processes (i.e., inflammation, cell death, proliferation, mitochondrial biology).
• redundant pathways and circuits.
• compensatory mechanisms.

Project-specific principles and criteria for the output prioritization can be related to the diseases or biology of interest, patient segments (e.g., genetic variants), specific therapeutic modalities or delivery routes, organs or tissues, or specific subcellular localization or process. Moreover, other factors such as existing standard of care, gaps in therapeutic approach and patient unmet needs, potential for combination with other products can help shape and guide the prioritization process. Implementation of the ranking strategy will represent cross-functional efforts between biologists, data scientists, and clinicians to create a prototype for a ranking algorithm, algorithm execution, and manual verification and curation of computer-based predictions.

### 4.3 Design—select and confirm drug candidates that have the highest potential to affect the intended MOA, utilizing a combination of experimental and computational models

For selecting drug components with a maximum effect on disease biology, experimental and computational models will have to be established for the top ranked MODs. Disease MOD can be represented by multiple *in vitro, ex vivo,* and *in vivo* models focused on specific phenotypes or pathways, especially in the context of multi-targeted combination design that may include effects on cell survival in multiple cell types, effect on biological processes requiring multi-cellular systems or *in vivo* models, e.g., fibrosis, or effect on complex phenotypes requiring specialized *in vivo* models, e.g., behavior and cognition. Once the models are established and phenotypes, timepoints, readouts and other features are optimized, these preclinical models will be used to validate predicted drug activity (MOA) against the multiple targets in the design (Haycock, 2011; Daou et al., 2021). The drugs and drug combinations selected in experimental and computational MOD models will be based on their ranks following prioritization driven by the key criteria relevant for the project. Special attention should be given to selection and characterization of biomarkers of pharmacodynamics and efficacy. Candidate biomarkers for evaluation in experimental systems can be identified based on the MOD/MOA networks and translational criteria including availability of reliable assays, ability for non-invasive measurements, and association of the biomarker changes to disease progression or efficacy in the clinic.

Computational or manual curation methods are applied to the MOD/MOA networks to determine whether individual compounds will be sufficient to achieve a maximum effect for each phenotype and experimental model or whether there is an optimal drug combination that is predicted to have a maximum effect. Top ranked predicted modulators are investigated for potential redundant, compensatory, inhibitory or activation properties. Simulations and computer modeling can be used for well-established systems with reliable parametrization to screen multiple drugs and drug combinations, with subsequent validation experimentally, and to bracket concentration ranges and relative ratios of combination composition, paving the way for optimization. The proposed drug design will include single drugs or drug combinations with experimentally and computationally confirmed, non-redundant, and complementary MOAs with a maximum effect on reversing the MOD.

### 4.4 Optimize—find the optimal composition, component ratios, and the dose to yield maximum treatment effect relevant for the clinical studies

In this step, the effect of drugs and drug combinations will be further evaluated in the experimental and computational models to determine the optimal drug characteristics, individual concentrations, and optimal dose. Translational modeling approaches, such as quantitative systems pharmacology (QSP, Azer et al., 2021; Bai et al., 2019; EFPIA MID3 Workgroup et al., 2016; Berg et al., 2005), are used to characterize the pharmacokinetic/pharmacodynamic (PK/PD) relationship and build the dose response curve ideally based on more than one preclinical model and measure the PD effect on multiple biomarkers to generate robust data. Optimal dose is usually selected to achieve maximum effect on selected PD biomarkers. To strengthen the confidence in translatability to the clinic, it is recommended to measure the same biomarkers in preclinical models that will be included in the clinical biomarker strategy. Experimental and computational models can also be utilized to evaluate the effect of genetic variants and epigenetic modifications relevant to disease of interest on dose, therapeutic effect, choice of biomarkers and other clinical trial design considerations.

In the optimization step, experimental design can be improved by computational models such as systems pharmacology and mechanistic modeling approaches (Zineh, 2019; EFPIA MID3 Workgroup et al., 2016; Bai et al., 2019) that can quantify the pharmacology of the composition and ultimately aid as a translation tool into the clinic to ascertain the clinical dose and quantify target engagement. QSP can also be applied with cell-based systems to quantify the contribution of individual components in the combination and aiding in addressing regulatory requirements like the fixed combination rule. QSP models, by integrating cellular and molecular interactions knowledge, support target and combination selection and validation in early drug discovery and optimal trial design (Cucurull-Sanchez et al., 2012; Benson et al., 2014; Betts et al., 2019; Chelliah et al., 2021) and provide a means of simulating differential response in genetically or mechanistically defined patient subtypes, as for the example in the case for SOD1-ALS (Paris et al., 2022). Data obtained via this process is modeled for drug exposure-response relationship using pharmacometrics techniques to demonstrate drug effects and dose optimization. “Fit-for-purpose” principles should guide decision-making and study planning based on the best pharmacological and biological knowledge (Kiyosawa and Manabe, 2016). The combination of preclinical and clinical data and computational models is subsequently utilized as we enter the clinical development phase to expand and advance understanding of the MOA, utilizing omics studies, metabolic experiments, flux studies, multicellular systems, among others.

### 4.5 Translate/roadmap to the clinic—develop a clinical path to inform clinical study design and biomarker strategy to validate pharmacology and efficacy in the clinic

Clinical development of novel drugs and drug combinations is dependent on non-clinical experiments and translational modeling to a significant extent (Yates et al., 2020). Successful translation is achieved by leveraging commensurate and precedented preclinical and clinical knowledge and data on disease endpoints, patient segments and biomarkers. Moreover, leveraging of natural disease history, real-world evidence and disease biomarkers studied in translational and clinical studies enables de-risking of later phase clinical trials and informs a strong clinical biomarker strategy. Designing and developing drug combinations (e.g., in rare diseases and oncology) is not only experimentally and clinically demanding. The predictability of disease biomarkers and endpoints from one trial to the other and *in vivo* to clinical translation remains an area of development for the field more broadly. Developing ‘virtual clinical trials’ with a ‘virtual population’ could be an opportunity to optimize chances of success and personalized treatment options for clinical practice (Kovatchev et al., 2009; Abrams et al., 2020). Moreover, PK/PD, QSP and translational modeling studies are critical in informing the dose and translational strategy for entry into the clinic (Haraya et al., 2022). Developing disease platform QSP models is a unique and powerful strategy to mitigate some of these challenges (Azer et al., 2021). In addition, utilizing systems biology analysis to identify genetic mutations or epigenetic changes linked to disease to help define patient subpopulations that are more likely to response to treatment is crucial. For example, understanding both genetically defined segments of Parkinson (such as GBA, LRKK2 and alpha-synuclein) and underlying MOD that may be overlapping across these segments, and potentially shared with other neurodegenerative diseases allows for optimal patient selection in POC trials focused on selected biomarkers relevant to the segment, as well as a longer term strategy of advancing combinations that may be beneficial across diseases with shared underlying biology (Merchant et al., 2019; Bloomingdale et al., 2022; Righetti et al., 2022).

Another emerging area with an enormous potential on improving clinical trial design with realistic duration to observe efficacy is collection of the real-world evidence and better understanding of natural disease history, and disease heterogeneity classifying patients based on their disease subtypes or disease progression rates. Since these efforts will lead to generation of large-scale multi-modal and rich, complex datasets, systems biology will be instrumental in data analysis and revealing of trends, signatures, and selection of patient subsets based on molecular biomarkers, demographic and clinical parameters who are more likely to respond to specific therapies based on the type of therapy or trial duration.

## 5 Challenges and future directions

While significant progress has been achieved over the last 2 decades and has revolutionized our view, understanding and opportunity to intervene for restoring health and maintaining wellness, several key challenges still lie ahead for the purpose of advancing innovative medicines to patients with unmet medical needs. We will differentiate here between two related types of challenges, namely, scientific and operational.

Some of the main areas of scientific challenge include elucidating mechanisms of disease for complex diseases that represent significant clinical heterogeneity such as neurodegenerative diseases, improving probability of success for translation to the clinic, and advancement in computational capability and commensurate data platforms to consolidate and synthesize vast and disparate sources of information and advance actionable information towards identification of novel targets and design of novel agents. We anticipate that progress in each of these areas will be incremental, and success dependent on effective industry-academic partnerships, expanded adoption and integration of multi-disciplinary research, as well as increased avenues of patient engagement in biomedical research.

Challenges in operationalizing systems biology in industry include balancing cost and resources for data generation and synthesis to maximize learning about disease processes for efficient drug design while staying on course in terms of budget and timelines. Moreover, strategic decisions around the balance of investment in platform capabilities to allow for speed, efficiency and scale, and application to specific programs through to the clinic and ultimately to approval are critical to developing a programmable at scale platform with timely and broad applications to biomedical science.

As progress unfolds across these and other areas of challenge, we expect to see future directions and opportunities unfold for systems biology and its applications in bringing innovative medicines to patient in need. The COVID-19 pandemic has shown programmable technology such as mRNA can rapidly be adapted to new applications, in this case a novel virus, and successfully vaccinate individuals and populations against emerging variants. Building on these successes, and advancements in RNA technology and CRISPR, systems biology will continue to be an invaluable tool in advancing and realizing the full potential of novel modalities like gene editing and RNA targeting in addressing previously unaddressable diseases. Moreover, broader and increased utility and application of systems biology in biotechnology research will be invaluable to stand up research and translational platforms that can fulfill the promise of biomedical research at scale by both improving pre-emptive health and restoring disease back to healthy homeostasis.

## 6 Summary

There is an unprecedented number of investigational products in clinical development for various diseases, including neurodegenerative, oncology, metabolic, cardiovascular, immune, rare, and neglected diseases. The complexity of the underlying biological systems is significant in these diseases and combinatorial therapies is increasingly taking centerstage. An urgent and imploring requirement exists for enhancing the process of efficient design and translation of drug candidates, and clinical trial design to improve the probability of success and reduce failure rates in the clinic. A proactive systems biology platform, that integrates computational and biological sciences, towards decoding complex and rare diseases with intricate biological networks is essential to enabling efficient and effective development of innovative medicines that impact unmet medical needs. Utilizing systems biology will enable the development of combination therapies designed for engineered MOD, and that provide novel insights into choosing the right treatment based on the disease types, and future adjacent opportunities in other disease areas. While this approach without a doubt will accelerate and improve drug discovery and development process for individual drugs, it is also more efficient and powerful for drug combinations that may be the answer for many complex diseases where the patient is left with little or no therapeutic options.

The drug development process is incrementally being adapted by biotech and pharmaceutical companies to increase efficiencies and improve probability of success by adapting to and deriving benefits from data-driven platform approaches that integrate the many facets and disciplines of drug discovery and development in a reproducible way and as part of an increasingly effective learning and confirming cycle. With the appropriate support from regulatory agencies, this will shape up as a standard practice wherein virtual trials are running ahead of and in parallel with clinical trials to accelerate the time for targeted treatment options for patients.

## Data Availability

The original contributions presented in the study are included in the article/Supplementary Material, further inquiries can be directed to the corresponding author.
